# Fexapotide triflutate vs oral pharmacotherapy as initial therapy for moderate-to-severe benign prostate hyperplasia patients: a cost-effectiveness analysis

**DOI:** 10.1186/s12894-022-01025-4

**Published:** 2022-05-13

**Authors:** Yifan Wei, Joel W. Hay, Alan R. Hay, Sze-chuan Suen

**Affiliations:** 1 Department of Pharmaceutical and Health Economics, School of Pharmacy, University of Southern California, 635 Downey Way, Los Angeles, CA 90089-3333 USA; 2 Kaiser Permanente Northwest, Portland, OR USA; 3 Daniel J Epstein Department of Industrial and Systems Engineering, Viterbi School of Engineering, University of Southern California, Los Angeles, CA USA

**Keywords:** Benign prostate hyperplasia, Cost-effectiveness analysis, Microsimulation model, Decision analysis

## Abstract

**Background:**

To assess the price range in which fexapotide triflutate (FT), a novel injectable, is cost-effective relative to current oral pharmacotherapy (5 *α*-reductase inhibitor, *α*-blocker, 5 α-reductase inhibitor and α-blocker combination therapy) as initial therapy followed by surgery for moderate-to-severe benign prostate hyperplasia patients with lower urinary tract symptoms (BPH-LUTS).

**Methods:**

We developed a microsimulation decision-analytic model to track the progression of BPH-LUTS and associated costs and quality-adjusted life years in the target population. The cost-effectiveness analysis was performed from Medicare’s perspective with a time horizon of 4 years using 2019 US dollars for all costs. The microsimulation model considered treatment patterns associated with nonadherence to oral medication and progression to surgery. Model parameters were estimated from large randomized controlled trials, literature and expert opinion. For each initial treatment option, simulations were performed with 1000 iterations, with 1000 patients per iteration.

**Results:**

Three upfront oral pharmacotherapy options are close in cost-effectiveness, with combination therapy being the most cost-effective option. Relative to upfront oral pharmacotherapy options, FT slightly increases quality-adjusted life years (QALY) per patient (1.870 (95% CI, 1.868 to 1.872) vs. 1.957 (95% CI, 1.955 to 1.959) QALYs). Under the willingness-to-pay (WTP) threshold of $150,000 per QALY, at price per injection of $14,000, FT is about as cost-effective as upfront oral pharmacotherapy options with net monetary benefit (NMB) $279,168.54. Under the WTP threshold of $50,000 per QALY, at price per injection of $5,000, FT is about as cost-effective as upfront oral pharmacotherapy options with NMB $92,135.18. In an alternative 10-year time horizon scenario, FT price per injection at $11,000 and $4,500 makes FT as cost-effective as oral pharmacotherapies. One-way sensitivity analysis showed this result is most sensitive to upfront therapy prices, FT efficacy and initial IPSS. At price per injections of $5,000, $10,000 and $15,000, the probability that FT is either cost-effective or dominant compared to upfront oral pharmacotherapy options using a WTP threshold of $150,000 per QALY is 100%, 93% and 40%, respectively.

**Conclusions:**

Compared to upfront oral pharmacotherapy options, FT would be cost-effective at a price per injection below $14,000, assuming a WTP threshold of $150,000 per QALY.

**Supplementary Information:**

The online version contains supplementary material available at 10.1186/s12894-022-01025-4.

## Background

Benign prostatic hyperplasia (BPH) is a chronic condition that is associated with progressive lower urinary tract symptoms (LUTS) and affects up to 50% of men over the age of 50 and up to 80% of men over the age of 80. As of 2010, BPH LUTS affects over 210 million men worldwide. Furthermore, BPH prevalence is on the rise, due to an increase in modifiable metabolic risk factors, such as obesity [1, 2].

BPH LUTS symptom severity can be evaluated by the International Prostate Symptom Score (IPSS) questionnaire (score of 0–7, mild; 8–19, moderate; 20–35, severe). Current treatment options for BPH LUTS include oral pharmacotherapy (*α*-blockers, 5-*α*-reductase inhibitors (5-ARIs), or combination therapy of *α*-blocker and 5-ARI) and surgeries such as transurethral resection of the prostate (TURP), which is the gold standard, and new surgery therapies (minimally invasive therapies, laser therapies and etc.) [3, 4]. According to the American Urological Association’s 2011 updated guideline on the management of benign prostatic hyperplasia, patients with persistent bothersome, moderate-to-severe symptoms can start with oral pharmacotherapy. Patients with still unresolved symptoms may elect surgeries [3]. Oral pharmacotherapy might have intolerable side effects and diminishing efficacy over time, and patients need to take them for the rest of their lives, which may lead to poor adherence. On the other hand, surgeries may expose patients to anesthetic risk and other adverse events such as incontinence, sexual dysfunction, stricture, etc.[3, 4]

Fexapotide triflutate (FT) is a first in-class compound given by local injection via the transrectal intraprostatic route under ultrasound guidance. Its efficacy and safety compared to placebo saline injection is supported by two placebo controlled double-blind randomized parallel group trials with 995 BPH patients with an average of 3.58 years of long-term follow-up [5]. Compared to oral pharmacotherapy and surgery, FT injection has many favorable aspects; it can relieve symptoms more effectively than oral pharmacotherapy, measured by a larger decrease in IPSS, and it does not have non-adherence issues as with oral pharmacotherapy [5, 6]. It also has a better adverse event profile compared with surgery. However, the cost of this novel injectable has not been established. A cost-effectiveness study can help identify the price range of FT that makes it as cost-effective as current oral pharmacotherapy as initial therapy followed by delayed surgery for moderate-to-severe BPH.

## Methods

### Strategies

We evaluated the cost-effectiveness of oral pharmacotherapy (i.e., 5-ARIs, *α*-blockers, 5-ARI + *α*-blocker) vs. FT as initial treatment followed by delayed surgery including transurethral resection of the prostate (TURP), Holmium laser enucleation of the prostate (HoLEP)/photoselective vaporization of the prostate (PVP) and UroLift prostatic urethral suspension implants for patients who failed the initial treatment. In total, four strategies were compared: (i) 5-ARIs followed by delayed interventional therapy; (ii) *α*-blockers followed by delayed interventional therapy; (iii) 5-ARI + *α*-blocker followed by delayed interventional therapy; (iv) FT followed by delayed interventional therapy. For details on surgery options included in the model, please refer to the Additional file 1: Technical Appendix.

## Target population

The target population is men with a mean age of 65 years, with moderate-to-severe BPH LUTS with no presumed contraindications. The target population in this model is to represent the patient population in FT’s clinical trial, NX02-0017 and NX02-0018. The mean baseline IPSS of this hypothetical patient cohort in our model is 23.5, with standard deviation of 4.96. In trial NX02-0017, patients’ baseline IPSS ranges from 12 to 35, the percentage of patients with baseline prostate volume less than 40 g in the FT treatment and placebo group are 50.3% and 51.0%, respectively; the percentage of patients with baseline urinary peak flow rate less than 5 mL/sec are 4.1% and 4.6%, respectively. In trial NX02-0018, patients’ baseline IPSS ranges from 15 to 35, the percentage of patients with baseline prostate volume less than 40 g in the FT treatment and placebo group are 46.2% and 45.6%, respectively; the percentage of patients with baseline urinary peak flow rate less than 5 mL/sec are 2.4% and 2.1%, respectively. [5] Simulated patients’ initial IPSS and change in IPSS under different treatment scenarios were randomly drawn from normal distributions with mean and standard deviation shown in Table1.

**Table 1:** Microsimulation model clinical input parameters affecting IPSS progression

IPSS	Mean (SD) (Standard deviation)	Distribution	Source
Initial IPSS	23.5 (4.96)	Normal	[5]
*IPSS progression per 3-month cycle*			
FT	− 3.00 (3.32)	Normal	[5]
Combination therapy, 1st cycle	− 4.80 (0)	-	[6]
Combination therapy	− 0.20 (0.21)	Normal	[6]
5-ARI, 1st cycle	− 2.80 (0)	-	[6]
5-ARI	− 0.31 (0.28)	Normal	[6]
α-blockers, 1st cycle	− 4.50 (0)	-	[6]
*α*-blockers	0.05 (0.19)	Normal	[6]
Natural, off treatment	0.045 (0.305)	Normal	[11]
Surgery effect on IPSS			
TURP, percent change in IPSS	0.27 (0.22)	Beta	[12]
PVP, IPSS value different from TURP	0.46 (2)	Normal	[12]
UroLift, percent change in IPSS	0.50 (0.34)	Beta	[13]
HoLEP, IPSS value different from TURP	− 0.78 (0.31)	Normal	[14]
No. of cycles	Lower/upper bound	Distribution	Source
*No. of cycles to reach maximum effect*			
FT	2/3	Uniform	[5]
Combination therapy	6/10	Uniform	[6]
5-ARI	6/10	Uniform	[6]
*α*-blockers	15/17	Uniform	[6]
No. of cycles effect lasts			
FT	16/31	Triangle	[5] and clinical expert opinion
TURP	16/31	Triangle	[10, 12]
PVP	16/31	Triangle	[10, 12]
UroLift	16/31	Triangle	[13]
HoLEP	16/31	Triangle	[14]

*IPSS* International prostate symptom score, *FT* Fexapotide triflutate, *5-ARI* 5-α-Reductase inhibitors, *TURP* Transurethral resection of the prostate, *PVP* Photoselective vaporization of the prostate, *HoLEP* Holmium laser enucleation of the prostate

## Outcomes

The outcomes evaluated were discounted costs, quality-adjusted life years (QALYs), and net monetary benefits (NMB). NMB is calculated as the product of an intervention’s incremental QALYs and the willingness-to-pay threshold, less incremental costs. Similar to incremental cost-effectiveness ratios (ICERs), NMB is also a measure to evaluate the cost-effectiveness of therapeutic interventions [7]. We choose to use NMB here since ICER can be hard to interpret when differences in QALYs are very small, and this is the case for our analysis.

## Cost-effectiveness analysis

The cost-effectiveness analysis was performed from Medicare’s perspective since the majority of patients affected by BPH LUTS are 65 and older and are covered by Medicare in the United States [8]. We used a time horizon of 4 years in the base case analysis; an alternative scenario with a 10-year time horizon was also evaluated as a robustness check for long-term outcomes. All future costs and benefits were discounted at 3% annually. Cost-effectiveness was determined using a conventional willingness-to-pay (WTP) threshold of $150,000 per QALY gained [9]; however, we also examined the effect of using a WTP threshold of $50,000 per QALY gained.

## Microsimulation model

A microsimulation decision-analytic model was developed in R statistical software version 3.6.3 (R Foundation for Statistical Computing, Vienna, Austria) to model the progression of BPH LUTS and to project the costs and QALYs of the target population [10]. Simulations were performed with 1000 patients per run for 1000 iterations for each initial treatment option in the base case 4-year and 10-year time-horizon scenarios and probability sensitivity analysis. In one-way sensitivity analyses, simulations were performed with 1000 patients per run for 100 iterations for each scenario. A microsimulation model was chosen to better track the IPSS and sequence of treatment procedures of each individual patient.

While population Markov models are popularly used for cost-effectiveness analyses, the individual-level heterogeneity and history-dependent treatment pathways inherent in BPH LUTS treatment was better modeled using a microsimulation, which could better capture the variation in initial treatment options and follow-up surgery procedures, variation in duration that the treatment effect will persist, and patients’ variation in adherence to oral pharmacotherapy. The advantage of using a microsimulation model is this information can be modeled and tracked easily.

**Fig. 1:**
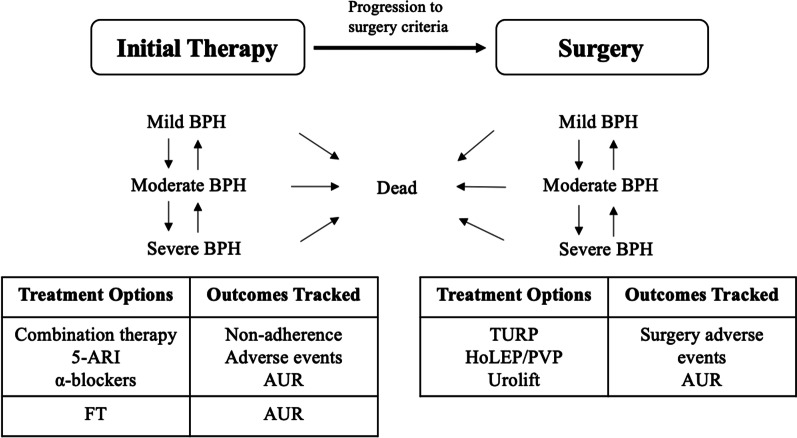
Conceptual model structure. BPH, benign prostate hyperplasia; 5-ARI, 5-*α*-reductase inhibitors; *FT* Fexapotide triflutate, *AUR* Acute urinary retention, *TURP* Transurethral resection of the prostate, *HoLEP* Holmium laser enucleation of the prostate, *PVP* Photoselective vaporization of the prostate

A cycle length of 3 months was used, following the practice of existing studies on similar topics and clinical experts’ opinion [11]. In each cycle of the model, an individual patient can improve or progress on IPSS score, discontinue treatment, progress to surgery, experience adverse events and BPH-related clinical outcomes, or die from other causes based on clinical probabilities associated with treatment options. They also accumulate costs and utility weights related to each event. Figure 1 shows a conceptual model structure diagram.

We made the following assumptions with regard to disease progression: (i) IPSS scores for patients on oral pharmacotherapy progress given the mean slope of IPSS change and its standard deviation for each oral pharmacotherapy option based on data from large randomized control trials comparing the efficacy of three oral pharmacotherapy options; off-medication IPSS progression is modeled based on natural history data from community dwelling men [6, 12]; (ii) patients who initiate treatment on FT will achieve a treatment effect of a 3 point average decrease in IPSS score with standard deviation 3.32 per cycle in 2 cycles, or a 6 point average decrease in IPSS score with standard deviation 6.64 in 1 cycle based on FT clinical trial data, and the effect will persist for 5–8 years; [5] (iii) patients that receive TURP achieved on average a 73% decrease in IPSS relative to their pre-procedure score, the treatment effect of HoLEP/PVP are relative to TURP; patients that receive UroLift achieved on average a 50% decrease in IPSS relative to their pre-procedure score; surgery procedures’ treatment effect will persist for 5–8 years [13–15]; (iv) after two years, three groups of patients will progress to surgery: (1) patients experienced twice acute urinary retention; (2) patients had a IPSS >  = 23; (3) patients had a IPSS between 15 to 23 and the IPSS score decrease from baseline is less than 3 points.

## Data sources

Model parameters including treatment efficacy, probability of adverse events and clinical outcomes were from large randomized controlled trials on BPH oral pharmacotherapy (CombAT study and MTOPS study), FT and BPH surgery procedures (GOLIATH study, L.I.F.T study) and existing BPH cost-effectiveness analysis [5, 6, 11, 13, 16, 17]. Some adverse events parameters related to sexual function came from systematic review papers [18, 19]. Adherence to oral medication parameters came from published work using Italian healthcare claims database, since work on the US population was not available [20, 21]. Age-specific mortality rate was from the 2017 US Life Table [22].

Utility values for mild, moderate, and severe BPH LUTS and disutility associated with adverse events and surgeries were from health-related quality of life literature and existing cost-effectiveness analysis studies on BPH LUTS patients [11, 23, 24]. An additive approach was taken to calculate combined utility values. Medicare as the payer’s perspective was adopted for this cost-effectiveness analysis. Drug costs were from the 2018 Medicare Part D drug spending data; procedure costs, which included surgery and management of adverse events, were from the 2018 Medicare Part B National Summary Data File, both price-adjusted to 2019 dollar using Medical CPI [25–27]. Service components of BPH diagnosis, treatments and management of adverse events were obtained from existing cost-effectiveness analysis on relevant topic [28]. Clinical input parameters affecting IPSS progression are shown in Table 1. For a detailed table of all model input parameters, please refer to the Additional file 1: Technical Appendix.

## Sensitivity and probabilistic analysis

Scenario analysis was conducted using 10-year time horizon for a robustness check for model results in the long-term. One-way sensitivity analysis was conducted to test model’s robustness to change in parameter values by increasing and decreasing parameters’ mean by 20%; parameters tested in one-way sensitivity analysis include initial IPSS of simulation cohort, FT’s efficacy, nonadherence rate to oral medications, adverse event rate for FT and prices of oral pharmacotherapies and FT. Probabilistic sensitivity analysis was performed to evaluate the model’s parameter uncertainty by having the same simulated patient (with assigned initial characteristics) going through two different initial treatment arms, with clinical and utility parameters randomly drawn each iteration. Probabilistic sensitivity analysis was conducted in the base case 4-year time horizon. Cost-effectiveness acceptability curve (CEAC) illustrated results from probabilistic sensitivity analysis. CEAC shows the probability one initial treatment option is cost-effective compared to its alternatives under different willingness-to-pay thresholds.

## Results

### Base case scenario

#### Cost outcomes

The costs, QALYS, and NMB results for all four strategies in the base case 4-year time horizon are shown in Table 2. Among oral pharmacotherapies, the strategy with upfront combination therapy is the least costly option at $1,308.58 per patients over 4 years, followed by *α*-blockers at $1,431.41 per patient and 5-ARI at $1,566.35 per patient. The costs for the upfront FT option depend on the price of FT and will be discussed later in the cost-effectiveness results.

**Table 2:** Costs, QALY and net monetary benefit results from base case 4-year time horizon

	Combination therapy	5-ARI	*α*-blockers	FT	
				Price $14,000	Price $5000
Costs per patient ($)	1308.58	1566.35	1431.41	14,831.46	5864.82
QALY per patient	1.87	1.87	1.87	1.96	1.96
Willingness-to-pay threshold at $150,000					
Net Monetary Benefit (NMB) ($)	279,191.42	278,633.65	279,068.59	279,168.54	288,135.18
Willingness-to-pay threshold at $50,000					
Net Monetary Benefit (NMB) ($)	92,191.42	91,833.65	92,068.59	83,168.54	92,135.18

*QALY* Quality-adjusted life years, *5-ARI* 5-α-Reductase inhibitors, *FT* Fexapotide triflutate

#### Quality adjusted life year (QALY) outcomes

The three upfront oral pharmacotherapy options provide the same 1.87 QALYs per patient over 4 years. The upfront FT option generates 1.96 QALYs per patient over 4 years, which is higher than all three upfront oral pharmacotherapy options.

#### Cost-effectiveness results

The NMB for the three upfront oral pharmacotherapy options are very close, as shown in Table 2. Upfront combination therapy is the most cost-effective option among the three upfront oral pharmacotherapy options; with a WTP threshold of $150,000 per QALY, its NMB is $279,191.42; with a WTP threshold of $50,000 per QALY, its NMB is $92,191.42. With a WTP threshold of $150,000 per QALY, FT’s NMB is $279,168.54 at price per injection of $14,000. At this price, FT is about as cost-effective as oral pharmacotherapies. The difference in NMB between FT and combination therapy is $22.88, or 0.008%. Under the WTP threshold of $50,000 per QALY, FT’s NMB is $92,135.18 at a price per injection of $5,000, making it about as cost-effective as oral pharmacotherapies, with only a $56.24 (or 0.06%) difference in NMB.

## Alternative scenario: 10-year time horizon

Costs, QALYs and cost-effectiveness results from 10-year time horizon scenario are shown in Table 3. In the 10-year time horizon scenario, combination therapy is still the most cost-effective option among upfront oral pharmacotherapy options with NMB $628,546.90 at WTP threshold of $150,000 per QALY and NMB 208,546.90 at WTP threshold of $50,000 per QALY. FT’s NMB is found to be lower than oral pharmacotherapies’ NMB in the 10-year time horizon scenario at price per injection of $14,000 (FT NMB $625,472.09 at WTP $150,000 per QALY) and $5,000 (FT NMB $207,932.19 at WTP $50,000 per QALY), which are the FT prices found in the base case analysis that makes it as cost-effective as oral pharmacotherapies. In the 10-year time horizon scenario, for a WTP threshold of $150,000 per QALY, FT’s NMB is similar to that of oral pharmacotherapies at $11,000 per injection (NMB $628,456.42). For a WTP threshold of $50,000, FT’s NMB is similar to that of oral pharmacotherapies at $4,500 per injection (NMB $208,429.03). This result shows that to make FT as cost-effective as upfront oral pharmacotherapy options, its price per injection needs to be lower in the 10-year time horizon than in the 4-year time horizon.

**Table 3:** Costs, QALY and net monetary benefit results from 10-year time horizon scenario

	Combination therapy	5-ARI	*α*-blockers	FT	
				Price $11,000	Price $4500
Costs per patient ($)	1453.10	1558.71	1571.75	12,043.58	5570.97
QALY per patient	4.20	4.20	4.20	4.27	4.28
Willingness-to-pay threshold at $150,000					
Net Monetary Benefit (NMB) ($)	628,546.90	628,441.29	628,428.25	628,456.42	636,429.03
Willingness-to-pay threshold at $50,000					
Net Monetary Benefit (NMB) ($)	208,546.90	208,441.29	208,428.25	201,456.42	208,429.03

*QALY* quality-adjusted life years, *5-ARI* 5-*α*-Reductase inhibitors, *FT* Fexapotide triflutate

## One-way sensitivity analysis

**Fig. 2:**
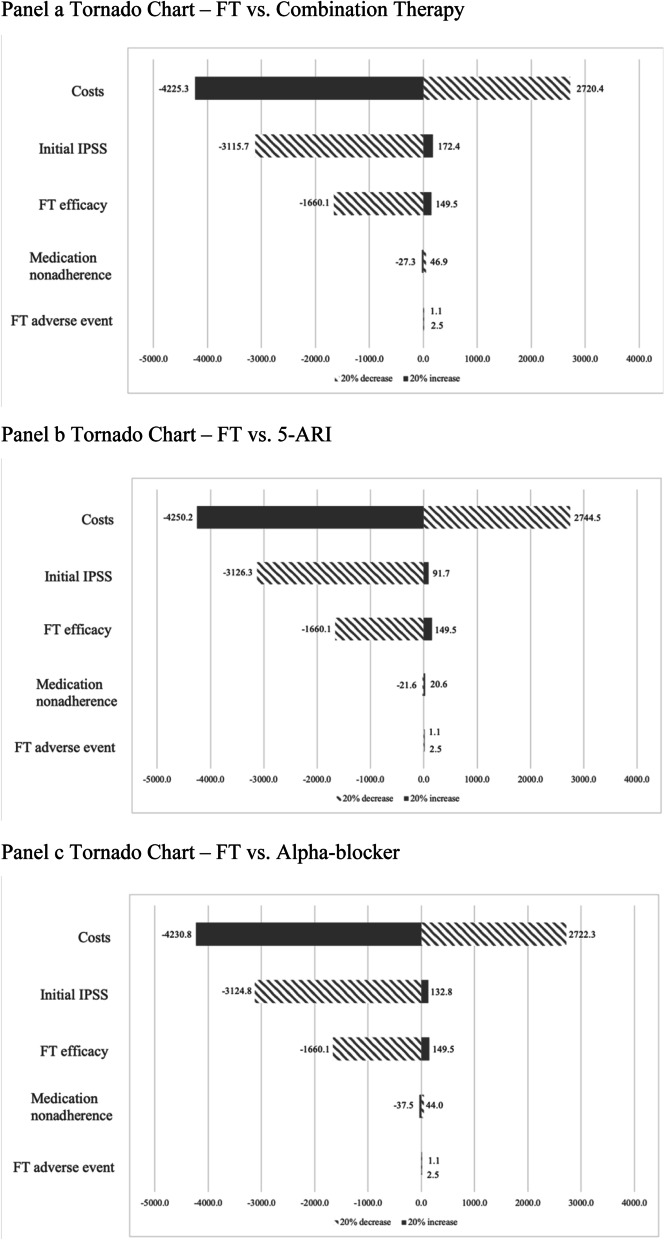
One-way sensitivity analysis tornado charts. *IPSS* International prostate symptom score *FT* Fexapotide triflutate, *5-ARI* 5-α-Reductase inhibitors

One-way sensitivity analysis results are shown in the tornado charts in Fig. 2. In one-way sensitivity analysis, we tested the robustness of results by adjusting the mean value by a 20% increase or decrease of the following parameters: initial IPSS of simulation cohort, FT’s efficacy, nonadherence rate to oral pharmacotherapies, adverse event rate for FT, and prices of oral pharmacotherapies and FT. We showed the net change in the differences between FT and oral pharmacotherapies’ NMB. One-way sensitivity analysis showed our result is most sensitive to prices of oral pharmacotherapies and FT, FT efficacy and initial IPSS. A 20% increase or decrease in the prices of upfront therapy heavily affects FT’s relative NMB. For example, when comparing to combination therapy, a 20% increase in the prices of both upfront therapies makes the differences in NMB between FT and combination therapy change from -$23 to -$4248, a net decrease of $4225. A 20% increase in FT’s efficacy or initial IPSS of the cohort will slightly increase FT’s cost-effectiveness relative to oral pharmacotherapies, while a 20% decrease in these two parameters will decrease FT’s cost-effectiveness much severely. The cost-effectiveness profile of FT is robust to changes in oral pharmacotherapies’ medication nonadherence rate and FT’s adverse event profile.

## Probabilistic sensitivity analysis

**Fig. 3:**
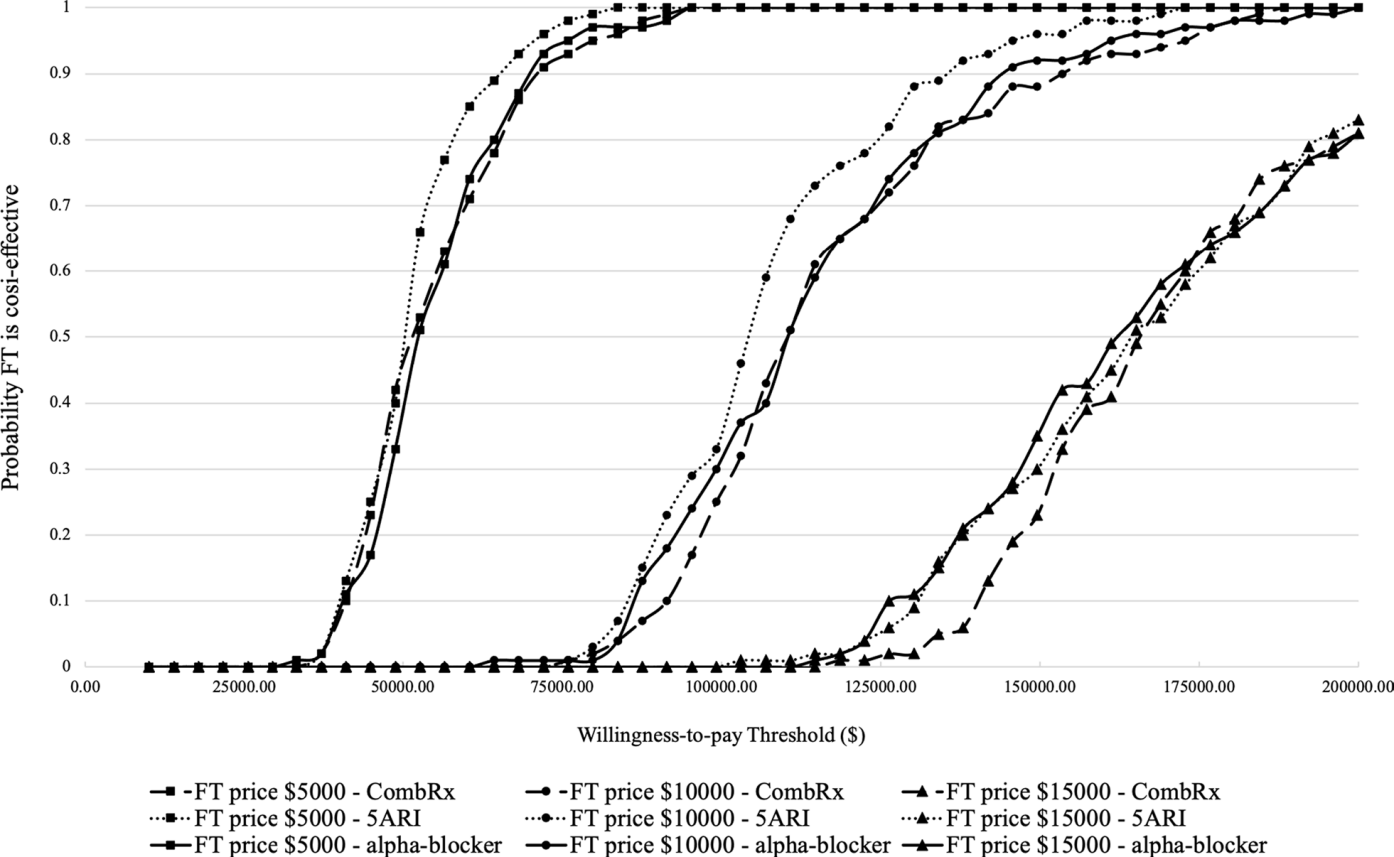
Cost-effectiveness acceptability curve of FT at different price with different willingness-to-pay threshold. *FT* Fexapotide triflutate; *5ARI* 5-α-Reductase inhibitors; *CombRx* Combination therapy

Figure 3 shows the cost-effectiveness acceptability curve of upfront FT compared to upfront oral pharmacotherapies, based on probabilistic sensitivity analysis results of FT at price per injection of $5,000, $10,000, and $15,000. At a price per injection of $5,000, FT’s probability of being cost-effective is near 100% when the WTP is over $75,000 per QALY. At a price per injection of $10,000, FT’s probability of being cost-effective is over 90% with WTP at $150,000. At a price per injection of $15,000, FT’s probability of being cost-effective is 40% with WTP at $150,000. FT’s cost-effective profiles are similar when compared to three oral pharmacotherapy options. At price per injections of $5,000, $10,000 and $15,000, the probability that FT is either cost-effective or dominant compared to oral pharmacotherapies using a WTP threshold of $150,000 per QALY is around 100%, 93%, and 40%, respectively.

## Discussion

This is the first study of the cost-effectiveness of fexapotide triflutate (FT), a novel injectable, to assess the price range in which FT is cost-effective relative to current oral pharmacotherapies on the market using microsimulation decision modeling. We compare FT to three oral pharmacotherapies, α-blockers, 5-ARI, and combination therapy, and find that at a price per injection of $14,000, FT is about as cost-effective as these oral pharmacotherapy options at a willingness-to-pay threshold of $150,000 per QALY. At price per injections of $5,000, $10,000, and $15,000, the probability that FT is either cost-effective or dominant compared to oral pharmacotherapies using a WTP threshold of $150,000 per QALY is around 100%, 93% and 40%, respectively.

Since our study is the first to evaluate the cost-effectiveness of FT, we are not able to compare its results on FT to other studies. Our results on oral pharmacotherapies are similar to previous cost-effectiveness analysis for BPH LUTS. Erman et al. used a microsimulation decision-analytic model to compare upfront oral pharmacotherapy followed by surgery upon failure and found combination therapy to be the most cost-effective in upfront oral pharmacotherapy options in a lifetime time horizon [11]. Using a state transition model to compare the three oral pharmacotherapy options followed by delayed TURP, Ismaila et al. also found combination therapy to be the most cost-effective option [29].

There are several limitations of our study. First, our model inputs on clinical effectiveness and adverse events were obtained from randomized trials. Though considered the ‘gold standard’ for measuring clinical effectiveness, randomized trial data may not be representative of patients in real-world practice. For example, adherence to long-term oral pharmacotherapy might be lower than reported in the trials, though we tried to account for this difference using real-world non-adherence data reported based on administrative claims database. Also, our study only focused on prescription medications as initial oral pharmacotherapy, though many over-the-counter medications, such as NSAIDs (nonsteroidal anti-inflammatory drugs), can also relieve BPH LUTS symptoms. Though the American Urological Association’s treatment guideline for moderate-to-severe BPH LUTS recommends patients to start with prescription medications as represented in our microsimulation model, in everyday practice over-the-counter medications could also be an important source of initial treatment for BPH LUTS patients. By focusing on prescription medications, our model follows the American Urological Association’s guideline but might not accurately represent what happens in the real world.

Second, since FT is a novel treatment, evidence regarding its effectiveness and adverse events profile are still limited in the literature. Our study uses information from its pivotal clinical trial combined with expert opinion. We also test our results’ robustness to FT’s effectiveness and adverse events profile in sensitivity analyses. Also, FT is reported in a more recent clinical trial to show long-term efficacy in the treatment of Grade Group 1 prostate cancer [30]. We did not account for FT’s potential efficacy for prostate cancer in this research since our study focused on BPH LUTS and the clinical trial for prostate cancer was conducted on a different patient population.

Furthermore, the choice of surgery options following upfront oral pharmacotherapy or FT treatment was limited in our model compared to surgery options available in the market. The decision of including TURP, PVP/HoLEP and UroLift in our model was made based on expert opinion and existing literature on surgery option prevalence in the market and relevant cost-effectiveness studies. Since the focus of our model is to compare the cost-effectiveness of FT and oral pharmacotherapies as initial therapy for BPH LUTS, we simplified the choice of surgeries while trying to include a representative list of options.

Our study also has several strengths. The use of a microsimulation model in this study enabled us to track individual-level heterogeneity in BPH LUTS patients like adherence to medication and secondary treatment options, since the treatment pattern of BPH LUTS is nonlinear. We obtained clinical input parameter for this microsimulation model from large BPH LUTS clinical trials, which ensures the quality of simulation outputs and comparability to other BPH LUTS cost-effectiveness analysis. Additionally, for parameters with high level of uncertainty, we performed one-way and probabilistic sensitivity analysis to test the results’ robustness, and we are able to show the probability of FT being cost-effective relative to different oral pharmacotherapies with different willingness-to-pay thresholds at different prices per injection.

## Conclusions

To our knowledge, this is the first study to assess the cost-effective price range of the novel injectable FT. Our study finds that from US Medicare’s perspective, for patients with moderate-to-severe BPH LUTS (with mean IPSS 23.5), using the novel injectable FT as initial therapy followed by delayed surgery is cost-effective compared to three current oral pharmacotherapy options at a price per injection of $14,000, with willingness-to-pay threshold of $150,000 per QALY. In this study, we only compared FT to oral pharmacotherapies as the upfront therapy followed by surgery. Future research could focus on the direct comparison of FT to surgery options as initial treatment for BPH LUTS, since FT has an attractive efficacy and adverse events profile and existing studies have found that upfront surgery treatment is more cost-effective compared to upfront oral pharmacotherapy options. Findings from our model can serve as a guide for future FT pricing. Since FT has not been on the market, this study can also be considered as a cost-effective price estimate for a BPH LUTS treatment with the same efficacy and adverse event profile as the FT injectable.

## Supplementary Information

**Additional file 1**. Technical Appendix.

## Data Availability

The datasets used and/or analysed during the current study are available from the corresponding author on reasonable request.

## Abbreviations

BPH: Benign prostatic hyperplasia
LUTS: Lower urinary tract symptoms
IPSS: International prostate symptom score
5-ARI: 5-*α*-Reductase inhibitors
TURP: Transurethral resection of the prostate
FT: Fexapotide triflutate
HoLEP: Holmium laser enucleation of the prostate
PVP: Photoselective vaporization of the prostate
QALY: Quality-adjusted life years
NMB: Net monetary benefits
ICER: Incremental cost-effectiveness ratios
WTP: Willingness-to-pay
CEAC: Cost-effectiveness acceptability curve
NSAIDs: Nonsteroidal anti-inflammatory drugs
AUR: Acute urinary retention
