# Supplementary material for: Fexapotide triflutate vs oral pharmacotherapy as initial therapy for moderate-to-severe benign prostate hyperplasia patients: a cost-effectiveness analysis
Source: BMC Urol. 2022 May 13;22:76. doi: 10.1186/s12894-022-01025-4 (PMC9102263; doi:10.1186/s12894-022-01025-4)
Supplement: Supplementary file 1 — Additional file 1. Technical Appendix. [file 12894_2022_1025_MOESM1_ESM.docx]

Fexapotide triflutate vs pharmacotherapy as initial therapy for moderate-to-severe benign prostate hyperplasia patients: a cost-effectiveness analysis

Technical Appendix

Choice of surgery options to include in the model

There are many surgery options available to manage BPH. TURP is still considered the gold standard, and other new therapies including laser therapies and minimally invasive therapies are increasingly available in recent years. Our goal in choosing surgery options to include in this model is to correctly represent the real-world landscape of surgical management of BPH without unnecessary nuances, since this study’s goal is to compare the cost-effectiveness of FT versus pharmacotherapy as initial, not subsequent interventional therapies. Based on literature review and expert opinion, we choose to include four surgery options in our model, which are transurethral resection of the prostate (TURP), Holmium Laser Enucleation of the Prostate (HoLEP), photoselective vaporization of the prostate (PVP) and Urolift.^1–3^ In our model, patients who need delayed surgery have equal probabilities, 1/3, of getting TURP, HoLEP/PVP and Urolift. We consider HoLEP and PVP, these two laser therapies, as one group; since based on clinical expert opinion, HoLEP uses increase and PVP uses decrease in recent years.

**Model parameter values and sources**

Pharmacotherapy options nonadherence probability (per 3-month cycle)

|  | 1yr | 2yr | 3yr | 4yr | 5yr | Reference |
| --- | --- | --- | --- | --- | --- | --- |
| Combination Therapy | 0.700 | 0.061 | 0.081 | 0.054 | 0.069 | [8] |
| 5-ARI | 0.580 | 0.040 | 0.040 | 0.060 | 0.050 | [8] |
| alpha-blockers | 0.410 | 0.040 | 0.050 | 0.070 | 0.040 | [8] |

Outcomes and Adverse Events (pharmacotherapy options 4-year outcomes, off treatment annual outcomes, surgeries and FT 3-month outcomes)

Lower 95% CI is 0.75 times of mean value, upper 95%CI is 1.25 times of mean value.

|  | Mean | Lower 95CI | Upper 95CI | Reference |
| --- | --- | --- | --- | --- |
| **Adverse Event** |  |  |  |  |
| **Dizziness** |  |  |  |  |
| Combination Therapy | 0.020 | 0.015 | 0.025 | [2] |
| 5-ARI | 0.010 | 0.0075 | 0.0125 | [2] |
| alpha-blockers | 0.020 | 0.015 | 0.025 | [2] |
| Natural, off treatment | 0.023 | 0.017 | 0.029 | [9] |
| **Gynecomastia** |  |  |  |  |
| Combination Therapy | 0.020 | 0.015 | 0.025 | [2] |
| 5-ARI | 0.020 | 0.015 | 0.025 | [2] |
| alpha-blockers | 0.010 | 0.0075 | 0.0125 | [2] |
| Natural, off treatment | 0.000 | 0.000 | 0.000 | [9] |
| **Ejaculatory dysfunction** |  |  |  |  |
| Combination Therapy | 0.030 | 0.023 | 0.038 | [2] |
| 5-ARI | 0.010 | 0.0075 | 0.0125 | [2] |
| alpha-blockers | 0.010 | 0.0075 | 0.0125 | [2] |
| Natural, off treatment | 0.008 | 0.006 | 0.010 | [9] |
| TURP | 0.112 | 0.107 | 0.115 | [10] |
| PVP | 0.066 | 0.056 | 0.076 | [10] |
| Urolift | 0 | 0 | 0 | [11] |
| HoLEP | 0.178 | 0.074 | 0.292 | [10] |
| **Erectile dysfunction** |  |  |  |  |
| Combination Therapy | 0.090 | 0.068 | 0.113 | [2] |
| 5-ARI | 0.070 | 0.0525 | 0.0875 | [2] |
| alpha-blockers | 0.050 | 0.0375 | 0.0625 | [2] |
| Natural, off treatment | 0.033 | 0.025 | 0.042 | [9] |
| TURP | 0.0084 | 0.0063 | 0.0107 | [10] |
| PVP | 0.0084 | 0.0063 | 0.0107 | [10] |
| Urolift | 0 | 0 | 0 | [11] |
| HoLEP | 0.0084 | 0.0063 | 0.0107 | [10], [12] |
| **Loss of libido** |  |  |  |  |
| Combination Therapy | 0.020 | 0.015 | 0.025 | [2] |
| 5-ARI | 0.030 | 0.0225 | 0.0375 | [2] |
| alpha-blockers | 0.020 | 0.015 | 0.025 | [2] |
| Natural, off treatment | 0.014 | 0.011 | 0.018 | [9] |
| **Urethral stricture or bladder neck contracture related to surgery** |  |  |  |  |
| TURP | 0.003 | 0.00225 | 0.00375 | [3] |
| PVP | 0.0179 | 0.0134 | 0.0223 | [3] |
| Urolift | 0 | 0 | 0 | [13] |
| HoLEP | 0.014 | 0.011 | 0.018 | [14] |
| **Bleeding** |  |  |  |  |
| TURP | 0.05 | 0.0375 | 0.0625 | [3] |
| PVP | 0.0402 | 0.0301 | 0.0502 | [3] |
| Urolift | 0 | 0 | 0 | [13] |
| HoLEP | 0 | 0 | 0 | [14] |
| **Blood transfusion** |  |  |  |  |
| TURP | 0.06 | 0.045 | 0.075 | [3] |
| PVP | 0.0268 | 0.0201 | 0.0335 | [3] |
| Urolift | 0 | 0 | 0 | [13] |
| HoLEP | 0 | 0 | 0 | [14] |
| **Outcomes** |  |  |  |  |
| **Incontinence** |  |  |  |  |
| Combination Therapy | 0.030 | 0.022 | 0.039 | [3] |
| 5-ARI | 0.037 | 0.028 | 0.046 | [3] |
| alpha-blockers | 0.040 | 0.031 | 0.050 | [3] |
| Natural, off treatment | 0.003 | 0.002 | 0.004 | [9] |
| TURP | 0.003 | 0.00225 | 0.00375 | [3] |
| PVP | 0.0670 | 0.0502 | 0.0837 | [3] |
| Urolift | 0.036 | 0.027 | 0.045 | [13] |
| HoLEP | 0.003 | 0.002 | 0.004 | [14] |
| **AUR** |  |  |  |  |
| Combination Therapy | 0.016 | 0.010 | 0.022 | [3] |
| 5-ARI | 0.023 | 0.016 | 0.030 | [3] |
| alpha-blockers | 0.051 | 0.040 | 0.062 | [3] |
| Natural, off treatment | 0.006 | 0.005 | 0.008 | [9] |
| TURP | 0.013 | 0.00975 | 0.01625 | [3] |
| PVP | 0.0670 | 0.0502 | 0.0837 | [3] |
| Urolift | 0.007 | 0.00525 | 0.00875 | [13] |
| HoLEP | 0.000 | 0.000 | 0.000 | [14] |
| FT | 0.0009 |  |  | [1] |

Utility Values (annual outcomes)

|  | Mean | Standard Deviation | Distribution | Source |
| --- | --- | --- | --- | --- |
| Mild BPH LUTS | 0.99 | 0.02 |  | [7] |
| Moderate BPH LUTS | 0.90 | 0.14 |  | [7] |
| Severe BPH LUTS | 0.79 | 0.11 |  | [7] |
| AUR | 0.82 | 0.18 |  | [7] |
| Dizziness | 0.91 | 0.10 |  | [7] |
| Ejaculatory dysfunction | 0.97 | 0.08 |  | [7] |
| Erectile dysfunction | 0.93 | 0.07 |  | [7] |
| Gynecomastia | 0.95 | 0.10 |  | [7] |
| Incontinence | 0.80 | 0.17 |  | [7] |
| Urethral stricture or bladder neck contracture related to surgery | 0.94 | 0.11 |  | [7] |
| TURP | 0.95 | 0.07 |  | [7] |
| PvP/HoLEP | 0.99 | 0.05 |  | [7] |
| Urolift | 0.99 | 0.05 |  | [7] |
| Baseline | 0.801 | 0.404 |  | [7] |

Costs (3-month for pharmacotherapy options and adverse events)

| Item | Costs | Source | Item | Costs | Source |
| --- | --- | --- | --- | --- | --- |
| alpha-blockers Initialization | 68.2 | [15] | AUR | 308.7 | [16] |
| 5-ARI Initialization | 65.4 | [15] | Incontinence | 419 | [16] |
| Combination Therapy Initialization | 120.5 | [15] | Erectile dysfunction | 47.3 | [16] |
| alpha-blockers | 54.0 | [15] | Ejaculatory dysfunction | 0 | [16] |
| 5-ARI | 51.3 | [15] | Gynecomastia | 0 | [16] |
| Combination Therapy | 106.3 | [15] | Dizziness | 0 | [16] |
| TURP | 1971.4 | [16] | Urethral stricture or bladder neck contracture related to surgery | 572.6 | [16] |
| Urolift | 2884.8 | [16] | Bleeding | 0 | [16] |
| HoLEP | 1925.1 | [16] | Blood transfusion | 35.4 | [16] |
| PVP | 1912 | [16] |  |  |  |

Reference

1. Shore N, Tutrone R, Efros M, et al. Fexapotide triflutate: results of long-term safety and efficiacy trials of a novel injectable therapy for symptomatic prostate enlargement. World Journal of Urology. 2018; 36: 801-809.
2. Roehrborn CG, Siami P, Barkin J, et al. The effects of combination therapy with 5-ARI and α-blockers on clinical outcomes in men with symptomatic benign prostatic hyperplasia: 4-year results from the CombAT study. European Urology. 2010; 57:123-131.
3. Thomas JA, Tubaro A, Barber N, et al. A multicenter randomized noninferiority trial comparing GreenLight-XPS laser vaporization of the prostate and transurethral resection of the preostate for the treatment of benign prostatic obstruction: two-yr outcomes of the GOLIATH study. European Urology. 2016; 69: 94-102.
4. Roehrborn CG, Gange SN, Shore ND, et al. The prostatic urethral lift for the treatment of lower urinary tract symptoms associated with prostate enlargement due to benign prostatic hyperplasia: the L.I.F.T study. The Journal of Urology. 2013; 190: 2161-2167.
5. Lei Y, Teng J, Huang CJ, et al. Holmium laser enucleation of the prostate versus transurethral resection of the prostate: a systematic review and meta-analysis of randomized controlled trials. Journal of Endourology. 2013; 27: 604-611.
6. Jacobsen SJ, Girman CJ, Guess HA, et al. Natural history of prostatism: longitudinal changes in voiding symptoms in community dwelling men. The Journal of Urology. 1996; 155:595-600.
7. Erman A, Masucci L, Krahn M, et al. Pharmacotherapy vs surgery as initial therapy for patients with moderate-to-severe benign prostate hyperplasia: a cost-effectiveness analysis. BJU International. 2018; 122: 879-888.
8. Cindolo L, Priozzi L, Soutoulides P, et al. Patient’s adherence on pharmacological therapy for benign prostatic hyperplasia (BPH)-associated lower urinary tract symptoms (LUTS) is different: is combination therapy better than monotherapy? BMC Urology. 2015; 15: 96.
9. McConnell JD, Roehrborn CG, Bautista OM, et al. The long-term effect of Doxazosin, Finasteride, and combination therapy on the clinical progression of benign prostatic hyperplasia. The New England Journal of Medicine. 2003; 349:2387-2398.
10. Frieben RW, Lin HC, Hinh, PP, et al. The impact of minimally invasive surgeries for the treatment of symptomatic benign prostatic hyperplasia on male sexual function: a systematic review. Asian Journal of Andrology. 2010; 12: 500-508.
11. McVary KT, Gange SN, Shore ND, et al. Treatment of LUTS secondary to BPH while preserving sexual function: randomized controlled study of prostatic urethral lift. J Sex Med. 2014; 11:279-287.
12. Zong HT, Peng XX, Yang CC, et al. The impact of transurethral procedures for benign prostate hyperplasia on male sexual function: a meta-analysis. Journal of Andrology. 2012; 33: 427-434.
13. Roehrborn CG, Gange SN, Shore, ND, et al. The prostatic urethral lift for the treatment of lower urinary tract symptoms associated with prostate enlargement due to benign prostatic hyperplasia: The L.I.F.T study. The Journal of Urology. 2013; 190:2161-2167.
14. Kuntz RM, Ahyai S, Lehrich K, et al. Transurethral holmium laser enucleation of the prostate versus transurethral electrocautery resection of the prostate: a randomized prospective trial in 200 patients. The Journal of Urology. 2004; 172:1012-1016.
15. Medicare Part D drug spending data. <https://www.cms.gov/Research-Statistics-Data-and-Systems/Statistics-Trends-and-Reports/Information-on-Prescription-Drugs/MedicarePartD.html>
16. Medicare Part B National Summary Data File. <https://www.cms.gov/Research-Statistics-Data-and-Systems/Downloadable-Public-Use-Files/Part-B-National-Summary-Data-File/Overview>
